# Identification and validation of lactate-related gene signatures in endometriosis for clinical evaluation and immune characterization by WGCNA and machine learning

**DOI:** 10.3389/fcell.2025.1672521

**Published:** 2025-10-07

**Authors:** Jixin Li, Xinya Hu, Caiyi Pan, Qing Liu, Siyang Zhang, Chiyuan Zhang, Xin Zhou

**Affiliations:** Department of Obstetrics and Gynecology, Shengjing Hospital of China Medical University, Shenyang, Liaoning, China

**Keywords:** endometriosis, lactate-related gene, diagnostic model, immune diversity, clinical management

## Abstract

**Background:**

Endometriosis is a common benign gynecologic disease in women of reproductive age, and its manifestations remarkably decrease quality of life. Lactate, as a metabolite, exerts prominent effects across a wide range of biological processes. The objective of this research is to explore the clinical value and immune features of lactate-related genes in endometriosis and contribute novel strategies for guiding the clinical management of patients with endometriosis.

**Methods:**

We first conducted a differential expression analysis to identify the differentially expressed genes (DEGs) in the training set. By integrating the critical module genes from weighted gene co-expression network analysis (WGCNA) and lactate-related genes (LRGs), we preliminarily screened lactate-related differentially expressed genes (LR-DEGs). Machine learning algorithms, single-cell datasets, and clinical samples were used to further identify and validate core LR-DEGs. Subsequently, we evaluated the diagnostic value of the model constructed from core LR-DEGs for endometriosis and explored the biological functions of these genes. Additionally, we conducted immune-related analysis in endometriosis and identified small molecule compounds targeting core LR-DEGs.

**Results:**

In this study, 22 candidate genes were identified by intersecting 2,318 DEGs and 2,177 key module genes with 357 LRGs. This list was further refined using three machine learning algorithms, resulting in three primary lactate-related biomarkers: BPGM, DHFR, and SLC25A13. A nomogram model constructed from core LR-DEGs demonstrated outstanding diagnostic performance in identifying patients with endometriosis. Immune-related analysis revealed significant associations between hub LR-DEGs and cellular immune dysregulation in endometriosis. Additionally, a gene–small molecule compound regulatory network was established to guide potential treatment strategies.

**Conclusion:**

Taken together, our study established a robust relationship between lactate metabolism-related genes and endometriosis, with the model promising to enable the early diagnosis of endometriosis, contribute to the excavation of the immune molecular mechanisms of endometriosis, and support the discovery of potential targets for therapy in the metabolism of endometriosis.

## 1 Introduction

Endometriosis is a common chronic inflammatory gynecological disease affecting approximately 5%–10% of all reproductive women worldwide and is characterized by the development of endometrial-like tissue outside of the uterus, which can induce symptoms such as chronic pelvic pain, dysmenorrhea, dyspareunia, and infertility in patients (Taylor et al., 2021; Zondervan et al., 2020). Currently, in the absence of clinical diagnostic markers capable of detecting or excluding endometriosis, laparoscopy, an invasive procedure, remains the gold standard for its identification. This limitation often leads to frequent misdiagnosis and substantially delayed diagnosis, sometimes by several years (Allaire et al., 2023; Becker et al., 2022). In addition, the lateness of such clinical diagnoses turns the practical management of endometriosis patients into the most challenging stage of medical advancement. In the present phase, the treatment of endometriosis is primarily focused on hormonal pharmacotherapy and operative therapies; although medication can partially alleviate patients’ pain, the duration of relief is limited, and the unavoidable side effects of drug administration make it intolerable for some patients. Surgery can also mitigate symptoms to some extent; however, it may alter the normal pelvic anatomical structure and promote pelvic adhesions, which have a certain impact on patients’ reproductive outcomes (Zondervan et al., 2020; Allaire et al., 2023; Marquardt et al., 2023). Moreover, approximately 50% of endometriosis patients will have a recurrence of the condition within 5 years, irrespective of the management options (Becker et al., 2017).

It is a fundamental characteristic of a cell that practically all biological activities necessitate the adjustment of its metabolic requirements in response to the alterations in the microenvironment (Lu et al., 2023). Glucose metabolism is the primary pathway for cellular energy production, comprising two major processes: glycolysis and oxidative phosphorylation. The Warburg effect refers to reprogramming of glycolytic metabolism, in which a cell, despite the availability of adequate oxygen, prefers the generation of lactate rather than utilizing the oxidative phosphorylation of glucose in the mitochondria to meet its energy requirements(Mao et al., 2022; Pavlova and Thompson, 2016). It has been demonstrated that this metabolic reprogramming procedure exists in patients with endometriosis in general, among whom such increased levels of glycolysis are closely associated with the development and advancement of endometriosis (Kasvandik et al., 2016; Lu et al., 2022). Concomitantly, there is a remarkable accumulation of lactate as a by-product of glycolysis in the peritoneal fluid of individuals with endometriosis, whereby it constitutes an environment that stimulates the migration and invasion of ectopic endometrial cells into the peritoneum and assists in the immunological escape that culminates in the formation of the endometriotic lesion (Young et al., 2014; Horne et al., 2019; Lefebvre et al., 2024). The concept of lactylation was first introduced in an article in the journal Nature, in which the term describes an epigenetic modification of lysine residues in histone proteins derived from lactate, which can directly participate in chromatin regulation for gene transcription (Zhang et al., 2019). Afterward, several studies have continuously expanded the understanding of lactylation and gradually unraveled its underlying mechanisms, demonstrating that lactylation not only regulates gene transcription by modifying histone proteins but also affects non-histone proteins, thereby governing a wide range of cellular processes, encompassing gene transcription, enzyme activity, protein stability, subcellular localization, protein interactions, and other post-translational modifications (Xu et al., 2025).

In light of the characteristics of endometriosis, especially its diagnostic challenges, treatment difficulties, and high recurrence rate, and considering the role of lactylation in cellular processes, we comprehensively explore the potential of lactate-related genes (LRGs) as biomarkers for endometriosis. We are committed to establishing a lactate-related gene diagnostic model to assist in the early diagnosis of endometriosis, while striving to elucidate the novel mechanisms of the disease from an immune microenvironment perspective and laying the foundation for achieving precision-targeted therapy for endometriosis.

## 2 Materials and methods

### 2.1 Data acquisition and processing

From the Gene Expression Omnibus database (GEO database, http://www.ncbi.nlm.nih.gov/geo/, accessed on 3 January 2025), clinical information and RNA expression data were retrieved from the endometriosis dataset GSE51981 (platform GPL570), which includes 77 clinical cases and 71 controls, and it was utilized as a training set due to its large sample size, which can present a robust substrate for model construction. Meanwhile, we searched two additional datasets for validation, namely, GSE7305 and GSE7307 (both from the platform GPL570), where the GSE7305 dataset consists of 10 endometriosis samples and 10 negative controls, while the GSE7307 dataset comprises 18 individual patients and 23 control cases. We merged and corrected the two aforementioned datasets using the “SVA” software package in an effort to create a unified dataset for the subsequent validation. Apart from that, the single-cell dataset GSE 213216 was screened for validation at the single-cell level, and two normal specimens and five ectopic endometrial samples from this dataset were selected for the subsequent analysis. From the Molecular Signatures Database (http://www.gsea-msigdb.org/gsea/index.jsp, accessed on 2 January 2025), we acquired a total of eight lactate-related gene sets and identified a total of 357 LRGs by eliminating duplicates.

### 2.2 Analysis of differentially expressed genes

To acquire differentially expressed genes (DEGs) between endometriosis and negative controls, we performed differential expression analysis using the “limma” R package (version 3.60.6) (Ritchie et al., 2015). The significance thresholds were set as adjusted *p*-value <0.05 and |log2 fold change (FC)| ≥ 0.5. The volcano plots plotted by the R package “ggplot2” (version 3.5.1) were utilized to visualize the results of the difference analysis. Afterward, boxplots were generated using the R package described above to visually represent the top 10 highly expressed genes and the least expressed genes.

### 2.3 Weighted gene co-expression network analysis

Weighted gene co-expression network analysis (WGCNA) is a systems biology approach used to identify co-expressed gene modules of high biological significance and examine correlations between gene networks and clinical features using the package “WGCNA” (version 1.73) (Langfelder and Horvath, 2008). The WGCNA package was used to analyze the training set based on disease severity characteristics. First, the top 25% of genes with the greatest variance were separated, and the Pearson correlation coefficients were computed to establish the adjacency matrix. This matrix employed a soft threshold power of 10 to ensure connection strengths aligned with the characteristics of a scale-free network. Then, the matrix was transferred into a topological overlap matrix (TOM) for a more comprehensive measure of similarity between the two genes, followed by the dynamic tree-cutting method to classify the modules, each of which contained a minimum of 30 genes, and the dynamic hybrid merging process to integrate the similar modules with a threshold of 0.25. Subsequently, modules were associated with clinical features by calculating gene significance (GS) and module correlation (MM), and ultimately, Pearson correlation coefficients were measured between combined modules and endometriosis, with the most strongly correlated module being considered the critical module to be selected for follow-up analysis.

### 2.4 Acquisition of lactate-related differentially expressed genes

To identify lactate-related differentially expressed genes (LR-DEGs) involved in endometriosis, we intersected the key module genes from WGCNA, DEGs, and LRGs. The results are visualized as a Venn diagram using the “ggvenn” package.

### 2.5 Functional enrichment analysis

Gene Ontology (GO) analysis and Kyoto Encyclopedia of Genes and Genomes (KEGG) pathway enrichment analysis are extensively available bioinformatics approaches, which are designed to systematically delineate the biological functions and pathways of genes. GO analysis annotates genes in terms of biological processes (BPs), cellular components (CCs), and molecular functions (MFs). Furthermore, KEGG pathway enrichment analysis is crucial for deriving the biological significance of transcriptome data. GO enrichment and KEGG pathway analyses of the target gene clusters were performed using the “clusterProfiler” toolkit (version 4.12.6), with statistical significance at *p*. adjust <0.05, and the appropriate R packages were utilized to visualize the meaningful results of GO and KEGG. Subsequently, the online website GeneMANIA (https://genemania.org) was used to demonstrate the protein interaction network of the core lactate-related genes and to construct a co-expression interaction network of these proteins.

### 2.6 Screening hub biomarkers using machine learning

To further optimize the identification of hub biomarkers, three machine learning algorithms were implemented for selecting the most representative and relevant target genes from the candidate genes. The least absolute shrinkage and selection operator (LASSO) logistic regression analysis was performed using the “glmnet” package, and non-essential genes were removed from the model by applying L1 regularization, which compresses the weights of unimportant genes to 0 (Simon et al., 2011). The support vector machine recursive feature elimination (SVM-RFE) is based on the SVM model for feature selection, and the significance of the features is ranked using the RFE model in an iterative manner (Sanz et al., 2018). The unimportant features are recursively eliminated to find the optimal subset of features using the “e1071” software package. In addition, the two aforementioned algorithms underwent 5-fold cross-validation, a resampling procedure (randomly dividing the data into five similar sizes, training with four subsets, validating with one subset, and repeating the process five times), and were used to evaluate the machine learning models for ensuring the stability and accuracy of the selected genes in different data groupings (Yang et al., 2023; Jiao et al., 2024). The random forest (RF) is a randomization algorithm that integrates multiple decision trees to significantly improve prediction performance, and the “randomForest” package was used to construct a random forest model, where the importance of each variable was evaluated to further select the top 10 variables (Degenhardt et al., 2019). Subsequently, the Venn diagram defined the key genes based on the overlap of the three machine learning results.

### 2.7 Evaluation and validation of essential genes

To evaluate the diagnostic efficacy of core targets and the nomogram, we utilized the “pROC” software package to calculate the area under the curve (AUC) of the receiver operating characteristic (ROC) curves, where values >0.6 indicate superior diagnostic performance. To assess the completeness of the validation dataset, the packages “FactoMineR” and “factoextra” were utilized to perform principal component analysis (PCA), and PCA plots were drawn to visualize the batch results. Subsequently, the expression levels of candidate genes in both the control and disease groups of the verification set were also determined and compared using a t-test between the two groups (*p* < 0.05) and illustrated in boxplots utilizing the “ggplot2” software package.

### 2.8 Single-cell database and gene expression analysis

To identify the expression patterns of core genes and the cell-communication landscape at the cellular level, we analyzed the single-cell dataset GSE 213216. The Seurat R package (version 4.3.0) was used for single-cell analysis. After performing quality control, we used the NormalizeData function to normalize the gene expression matrix. The Harmony method was applied to integrate data from different samples and mitigate batch effects. Based on the marker genes, all cells in the seven samples were classified into seven cell types. The DimPlot function was used to visualize the cells according to their cell types in the t-distributed stochastic neighbor embedding (tSNE) space, and the VlnPlot function was used to display the expression patterns of genes across different cell types. The CellChat R package (version 1.6.1) was used to infer cell communication roles and establish communication patterns between the cells.

### 2.9 Construction of the predictive nomogram

A nomogram is a widely used data visualization tool in medical research for predicting the occurrence of clinical events on an individual basis. To forecast the risk of endometriosis, a nomogram comprising specific key genes was derived and mapped using the “rms” package, where each variable is attributed a respective score in a nomogram scoring system, and the aggregate of the individual scores for all the genes per patient represents the total score, which is computed to estimate the endometriosis risk for patients. Moreover, decision curve analysis (DCA) was utilized to evaluate the clinical utility of the nomogram model, along with the generation of calibration profiles and ROC curves to assess the predictive accuracy of the model (Ngiam and Khor, 2019).

### 2.10 Gene correlation analysis

Gene correlation analysis is a critical bioinformatics technique for estimating the correlation of gene expression patterns, which reveals the underlying functional linkages or regulatory relationships among the genes. In order to detect the expression connections among the three critical genes, the Pearson correlation analysis was carried out using the “correlation” R package, followed by visualization using the “ggplot2” R package.

### 2.11 Gene set enrichment analysis

To explore the regulatory pathways and biological attributes in which the hub genes were likely to be implicated, a single gene set enrichment analysis of the shortlisted key genes subjected to different expression conditions was carried out using the “clusterProfiler” package (version 4.12.6), setting the threshold at *p*. adjust <0.05 (Mootha et al., 2003). Subsequently, the first five significantly enriched KEGG pathways were selected for visualization.

### 2.12 Consensus clustering analysis

Consensus clustering is an integrated method of identifying stable and credible aggregates from multiple clustering outputs (Zhang and Dai, 2024). To determine stable molecular subtypes and evaluate the robustness of the clusters, a consensus clustering analysis was carried out on the clinical samples within the training set using the R package ConsensusClusterPlus (version 1.68.0). For stability and reliability of the clustering results, 1,000 iterations were executed to produce a massive sub-dataset, and each of them was clustered using the “k-means” algorithm in accordance with the 80% resampling ratio to constitute a consensus matrix (Wilkerson and Hayes, 2010). Finally, the optimum clustering number was set to K = 2, in accordance with the cumulative distribution function (CDF) curve of the matrix, and the outcomes were presented using heatmaps, CDF plots, and incremental area plots.

### 2.13 Immune cell and immune function analysis

CIBERSORT (https://cibersort.stanford.edu/) is a pioneering bioinformatics tool for predicting the comparative abundance of various immune cell types among samples based on the gene expression data using a deconvolution algorithm (Newman et al., 2015). Using “CIBERSORT” (version 0.1.0), samples from both sub-clusters were subjected to immune infiltration analysis, which quantified the relevant abundance of 22 immune cell types in the context of endometriosis. This was followed by a comparison of immune cell infiltration levels between the two sub-clusters, with the results visualized graphically using boxplots generated by “ggplot2.” Subsequently, the “estimation” package was utilized to summarize, calculate, and compare the immune, stromal, and ESTIMATE scores between the two subgroups, and the respective boxplots were generated to present the outcomes more clearly (Yoshihara et al., 2013). The single-sample gene set enrichment analysis (ssGSEA) algorithm was used to parse 13 immune functions for immune correlation analysis, while related boxplots and heatmaps were produced using the “ggplot2” and “pheatmap” packages, respectively, to demonstrate the infiltration differences of the immune functions (Bindea et al., 2013). Simultaneously, 28 immune cells and three signature genes were subjected to Pearson correlation analysis to elucidate their relationships using the abovementioned package, and the outcomes were visualized using the mapping package (Jiang et al., 2021).

### 2.14 Organizing the core gene regulatory networks

NetworkAnalyst (https://www.networkanalyst.ca/NetworkAnalyst/) is a comprehensive biological network analysis and visualization platform containing a large number of databases for analyzing and understanding bionetworks (Lei et al., 2025). Data on miRNAs and transcription factor regulators were extracted from the online websites for the upstream prediction of core DEGs. Afterward, the candidate gene regulatory networks were optimally visualized within Cytoscape software. To discover potential therapeutic targets for endometriosis, CTD (http://ctdbase.org/) was used to predict small-molecule drugs directed against candidate genes, based on which a network of gene–drugs was developed for treatment applications.

### 2.15 RNA isolation and quantitative reverse transcriptase PCR

The methodology for this section was as described in our previous article; in brief, total tissue and cellular RNA were extracted from the control and endometriosis groups, followed by cDNA synthesis and qRT-PCR analysis in accordance with the provided instructions (Li et al., 2023). Likewise, actin was selected as the internal reference gene, and the data were analyzed utilizing the 2^−ΔΔCt^ method. The primer sequences of the related genes are presented in Supplementary Table S1.

### 2.16 Isolation, culture, and characterization of endometrial stromal cells

As previously reported in our study, fresh tissues were obtained from the operating room, transferred to the laboratory as soon as possible, washed with PBS, digested with type IV collagenase, and sieved to obtain normal endometrial stromal cells (NESCs) and ectopic endometrial stromal cells (EESCs), which were subsequently cultured in an incubator at 37 °C and 5% CO_2_. The cells were then identified by immunofluorescence, with vimentin used as a positive marker for stromal cells and E-cadherin serving as a negative control for epithelial cells.

### 2.17 Statistical analysis

Statistical analyses in this study were primarily performed using R software(version 4.3.1) and the associated R packages, and *p*-values <0.05 were considered statistically significant, with the degree of significance indicated as follows: * if *p* < 0.05, ** if *p* < 0.01, and *** if *p* < 0.001.

## 3 Results

### 3.1 Screening of common DEGs and analysis of functional roles

**FIGURE 1:**
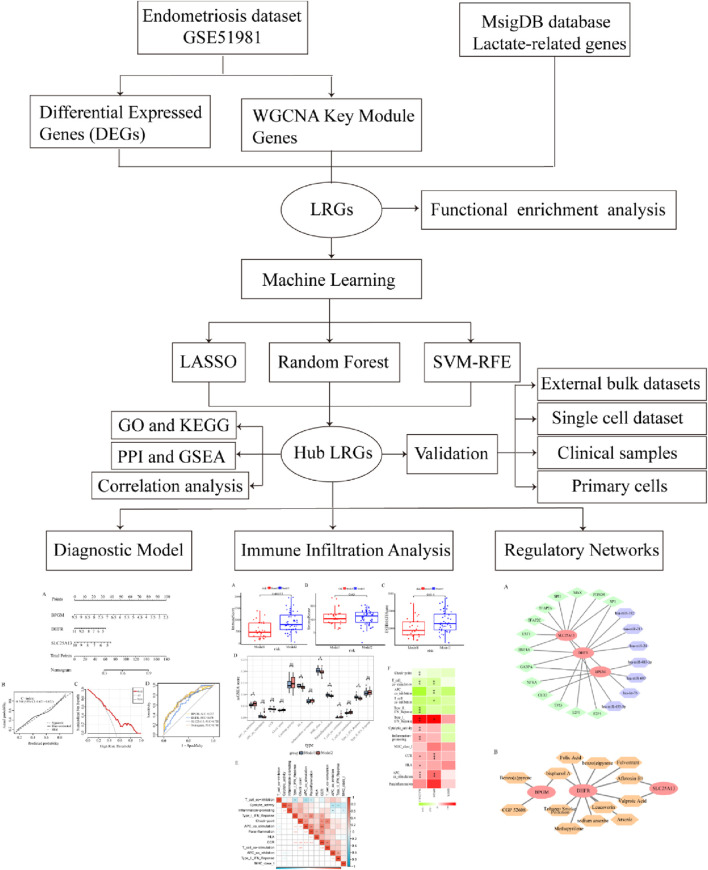
Flowchart of the elaborative study.

**FIGURE 2:**
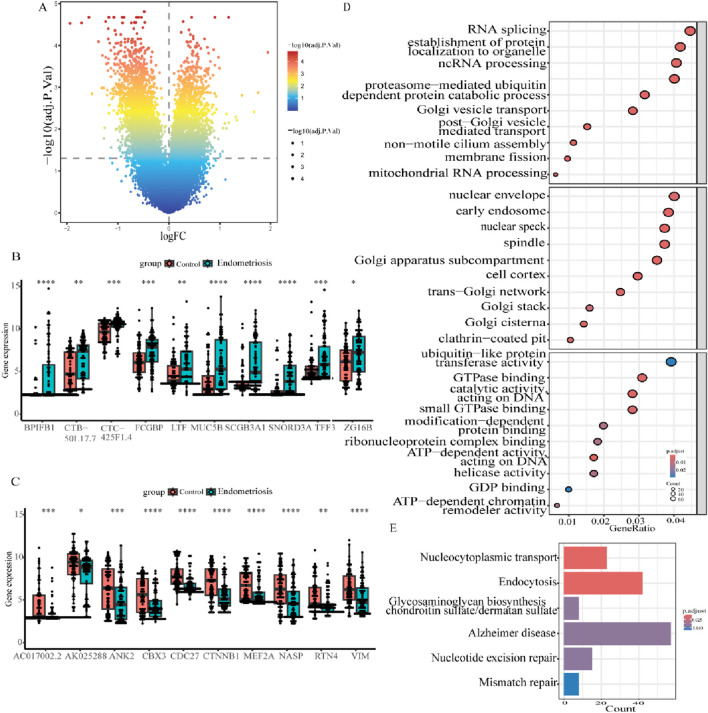
Identification and profiling of DEGs in endometriosis. **(A)** Volcano diagram of differentially expressed genes in GSE51981. **(B, C)** Boxplots illustrating the expression of the top 10 up- and downregulated genes in GSE51981. **(D)** Bubble plots of the first 10 BPs, CCs, and MFs in GO enrichment analysis. **(E)** Results of KEGG analysis for DEGs in the bar plot (*p < 0.05, **p < 0.01, ***p < 0.001, and ****p < 0.0001).

A flow diagram of the entire research is shown in Figure 1. Initially, as per the adjusted *p*-value <0.05 and |log2 fold change (FC)|≥0.5, a total of 2,318 DEGs between the endometriosis group and the control group were identified from the dataset GSE51981, wherein 591 were upregulated genes and 1,727 were downregulated genes, as displayed in volcano plots (Figure 2A). Subsequently, boxplots illustrated the expression profiles of the top 10 up- and downregulated genes (Figures 2B, C). After that, these DEGs were subjected to GO and KEGG enrichment analyses to further investigate their associated biological functions and pathways. The GO analyses revealed that a high percentage of genes were involved in the biological processes of “RNA splicing,” “establishment of protein localization to organelle,” “ncRNA processing,” and “proteasome-mediated ubiquitin-dependent protein catabolic process.” With respect to the cellular component, such genes were mostly associated with the “nuclear envelope,” “early endosome,” and “nuclear speck.” The molecular functions in which DEGs were significantly enriched included “ubiquitin-like protein transferase activity,” “GTPase binding,” and “DNA-dependent catalytic activity” (Figure 2D). It was observed in KEGG analysis that a significant enrichment of the genes under research was found in several enrichment pathways, namely, “Alzheimer’s disease,” “endocytosis,” and “nucleocytoplasmic transport” (Figure 2E). These findings indicated that the DEGs in individuals with endometriosis are mainly involved in gene expression processes, transport procedures, and energy metabolism-related activities.

### 3.2 Implementation of WGCNA to acquire essential module genes

**FIGURE 3:**
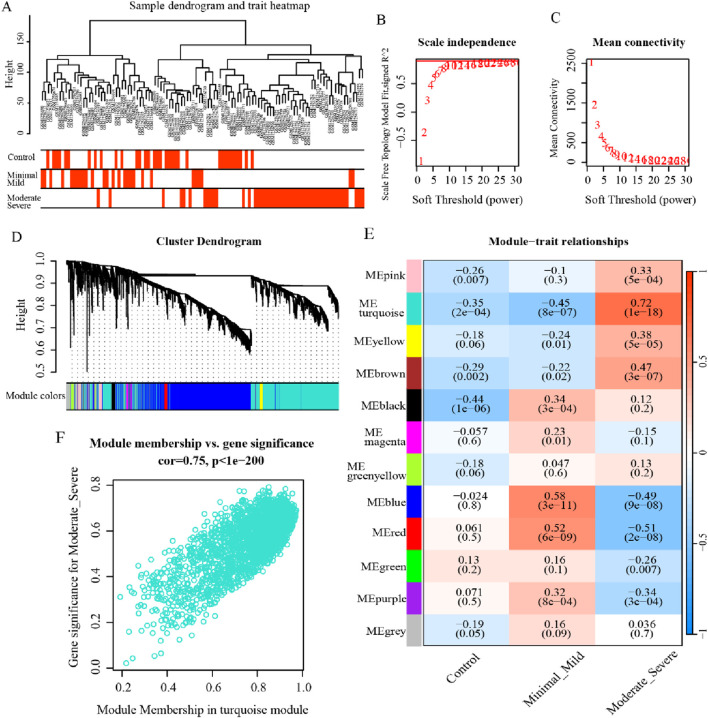
WGCNA for the critical modules associated with endometriosis. **(A)** Dendrogram and heatmap displaying the hierarchical clustering results of 148 samples. **(B, C)** Scale-free connectivity index and average connectivity analysis outcomes under different soft thresholds. **(D)** Gene cluster dendrogram and network heatmap for endometriosis by dynamic tree-cut algorithm. **(E)** Heatmap illustrating the correlation between modules and clinical traits, where the turquoise-colored module was strongly correlated with endometriosis severity (*p* < 0.05). **(F)** The scatterplot for the turquoise-colored module displays the relationship between module membership and gene significance (*p* < 0.05).

In an effort to further recognize the gene modules most associated with the clinical characterization of endometriosis, WGCNA was executed on the dataset GSE51981. The sample clustering was revealed to be solid, which implies that no outlier cases were excluded in the subsequent analysis (Figure 3A). Then, the outcomes of the scale-free connectivity index and average connectivity analysis show that when the soft threshold β = 10, the unscaled fit index increases, with the signed *R* ^2^ approaching the crucial level of 0.9, and the average connectivity tapers to 0, at which point the network approximates a scale-free distribution (Figures 3B, C). Based on the optimal soft threshold, the correlation matrix was converted to an adjacency matrix, followed by the TOM to detect the gene modules relevant to the severity of endometriosis for subsequent module delineation. After that, the dynamic tree-cutting method was applied to categorize the modules and merge the similar ones, resulting in 12 different modules (Figure 3D). Out of these, the turquoise-colored module exhibited the most significant correlation with endometriosis severity (r = 0.72, p = 1e-18) (Figure 3E). Accordingly, 2,177 genes in the module were identified as critical module genes for further analysis (Figure 3F). Taken together, these findings provide valuable insights into gene expression changes associated with endometriosis and highlight the relevance of the ME turquoise-colored module with respect to disease severity.

### 3.3 Identification and functional enrichment analysis of lactate-related DEGs

**FIGURE 4:**
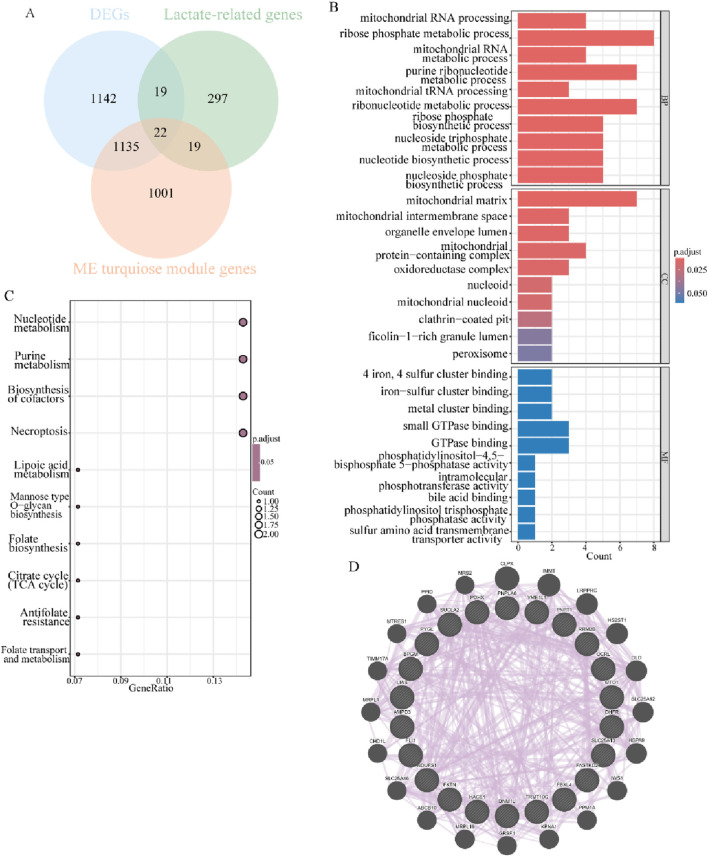
Characterization and functional analysis of LR-DEGs related to endometriosis. **(A)** Venn diagram revealing intersected DEGs, key modular genes, and LRGs to obtain 22 LR-DEGs. **(B)** Bar graphs for GO analysis of LR-DEGs (*p*.adjust <0.05). **(C)** Bubble plots showing the results of KEGG analysis of LR-DEGs (*p*.adjust <0.05). **(D)** PPI network of mutual interactions across 22 LR-DEGs.

Overlapping the 2,318 common DEGs, 2,177 key module genes, and 357 lactate-related genes in endometriosis mentioned above, 22 lactate-related differentially expressed genes were obtained (Figure 4A). To explore the functional effects of these 22 LR-DEGs in the context of the studied condition, they were subjected to further GO and KEGG enrichment analyses. The findings of the GO analysis revealed that these LR-DEGs were mainly engaged in the ribose phosphate metabolic process, purine ribonucleotide metabolic process, and ribonucleotide metabolic process, and they were apparently localized in the mitochondrial matrix (Figure 4B). Nucleotide metabolism, purine metabolism, and biosynthesis of cofactors were the significantly enriched pathways with important roles, as detected through KEGG analysis (Figure 4C). Additionally, the protein–protein interaction relationships of such LR-DEGs indicated that they can share “CLPX,” “IMMT,” and “LRPPRC” with more comprehensive protein interactions, which may be critical in biological processes (Figure 4D). All of the aforementioned results suggested that the LR-DEGs were intimately associated with energy metabolism activities and, remarkably, highlighted the influence of lactate-related elements on the progression of endometriosis.

### 3.4 Characterization of core lactate-related DEGs by machine learning

**FIGURE 5:**
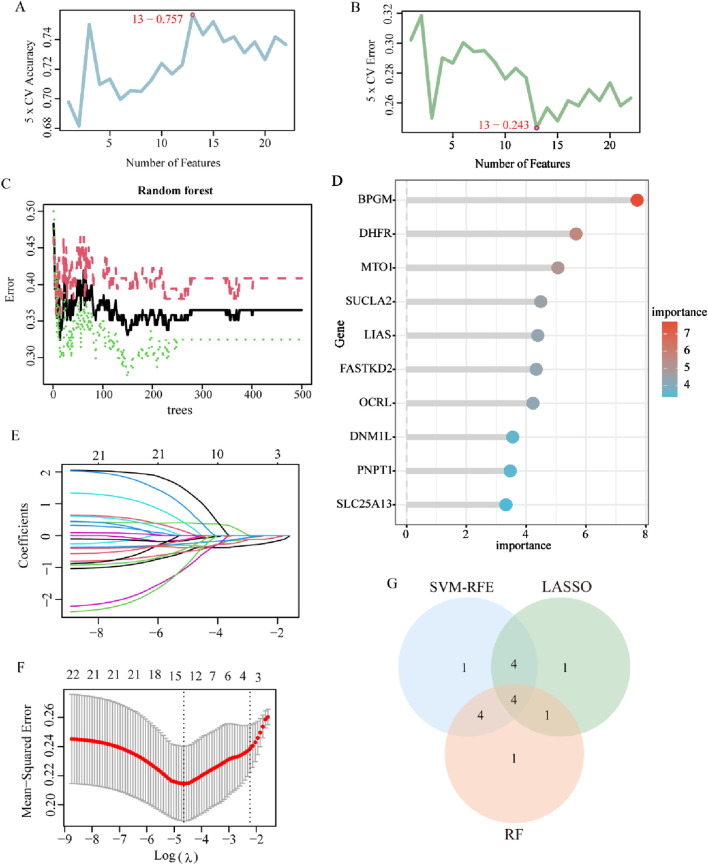
Machine learning for screening of lactate-related biomarkers in endometriosis. **(A, B)** Curve of change in the predicted accuracy and error value in the SVM-RFE algorithm (*p* < 0.05). **(C)** Correlation between the total number of trees and the error rate based on the random forest algorithm (*p* < 0.05). **(D)** Top 10 genes in ranking the relative importance scores in RF. **(E, F)** LASSO regression algorithm to extract key LR-DEGs (*p* < 0.05). **(G)** Venn diagram showing the four key LR-DEGs identified by the intersection of the three machine learning algorithms.

Aiming to further identify key genes with greater clinical significance, 22 LR-DEGs were assessed and analyzed utilizing three machine learning algorithms, namely, LASSO, SVM-RFE, and RF. At first, they were screened using the SVM algorithm, which revealed that the root mean square error (RMSE) reached the lowest value when collecting a total of 13 genes, which were maintained as the critical genes (Figures 5A, B). Later on, using the RF model, the first 10 genes on the importance scale were prioritized (Figures 5C, D). In addition, with the application of the fivefold cross-validated LASSO regression algorithm, the top 10 characterized genes were appraised (Figures 5E, F). Eventually, by crossing over the results of these three algorithms, four core LR-DEGs were determined, which are BPGM, DHFR, SLC25A13, and FASTKD2 (Figure 5G).

### 3.5 Evaluation and verification of crucial LR-DEGs in the relevant data

**FIGURE 6:**
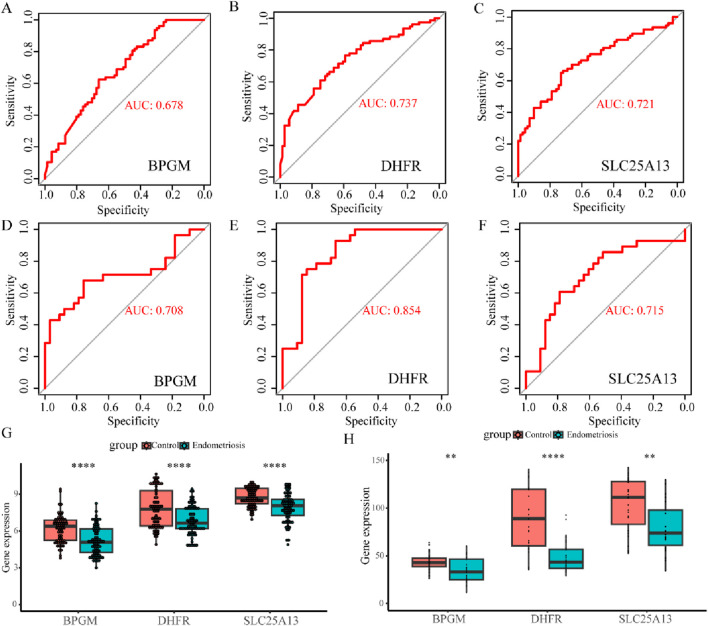
Value and verification of crucial LR-DEGs in the external databases. **(A–C)** ROC curves for BPGM, DHFR, and SLC25A13 in GSE51981. **(D–F)** ROC analysis results of three biomarkers in the validation set. **(G, H)** Differences of expression levels for BPGM, DHFR, and SLC25A13 between the control and disease groups in the training and validation sets (*p < 0.05, **p < 0.01, ***p < 0.001, and ****p < 0.0001).

To further evaluate the diagnostic precision of BPGM, DHFR, SLC25A13, and FASTKD2, the four candidate genes were subjected to ROC analysis, and the results indicated that BPGM (AUC = 0.678), DHFR (AUC = 0.737), and SLC25A13 (AUC = 0.721) demonstrated satisfactory diagnostic performances (Figures 6A–C). On account of the poor diagnostic accuracy of FASTKD2 (AUC <0.6) (Supplementary Figure S1), it was not considered for follow-up examinations. Following this, we also assessed the efficacy of these three core genes in the identification of endometriosis in the validation set, and the outcomes were rigorously validated, with the respective AUC values of BPGM, DHFR, and SLC25A13 all being above 0.7, among which the maximum value of 0.854 was achieved for DHFR (Figures 6D–F). Afterward, the expression levels of the three key LR-DEGs derived from filtering in the training set were assayed between the endometriosis and control groups. In the results, BPGM, DHFR, and SLC25A13 were remarkably lower expressed in the disease group, with statistically significant differences (Figure 6G). Similarly, the expression profiles of these genes in the validation datasets GSE7305 and GSE7307 after eliminating the batch effect yielded an identical result with the same trend (Supplementary Figure S2; Figure 6H).

**FIGURE 7:**
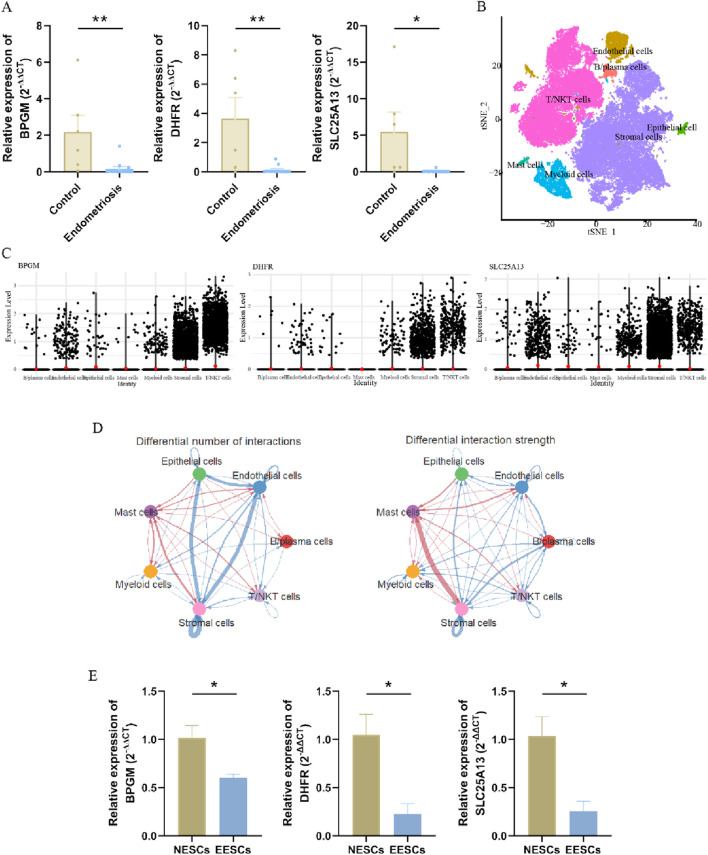
Validation of three hub LR-DEGs in the clinical samples. **(A)** Expression levels of BPGM, DHFR, and SLC25A13 validated in clinical tissues. **(B)** Visualization of the different cell clusters in the UMAP. **(C)** Violin plot displaying the expression of three key core genes in the different cell groups. **(D)** Circular diagram representing cell–cell communication, indicating the number and strength of the seven cell types interacting with each other in endometriosis. **(E)** Verification of the expression levels of BPGM, DHFR, and SLC25A13 at the cellular level (*p < 0.05 and **p < 0.01).

To fully confirm the accuracy of our findings, we also measured the expression of these three genes in terms of clinical samples, which, as expected, showed the aforementioned tendency (Figure 7A). Then, we proceeded further with the validation in the single-cell dataset, which was categorized into seven cell clusters based on the cellular classical markers, and it revealed that these three genes were mainly expressed in the stromal cells’ group; therefore, we carried out verification again at the cellular level (Figures 7B, C). In addition to analyzing gene expression profiles, we utilized the advantages of single-cell datasets to further evaluate interactions between cells. As shown in Figure 7D, the stromal cells exhibited the highest number of interactions with endothelial cells, while the strength of interactions between stromal and mast cells was the most pronounced. Subsequently, primary endometrial stromal cells were extracted and characterized, and the mRNA expression of three hub genes was evaluated, showing that their expression trend was identical to that of the training set (Supplementary Figure S3; Figure 7E). Therefore, the lactate-related biomarkers BPGM, DHFR, and SLC25A13 were differentially expressed in endometriosis, and we clarified the primary cell types where these genes were located and the potential cell-to-cell interactions through which these core genes function in endometriosis.

### 3.6 GSEA and correlation analysis of hub genes

**FIGURE 8:**
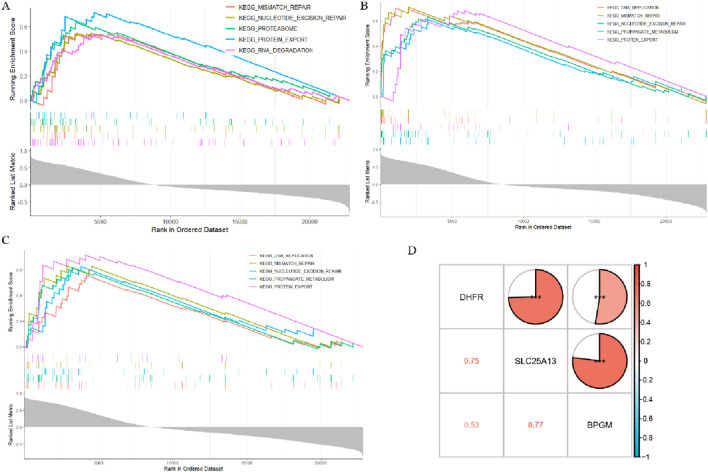
Biological function and correlation analysis of LR-DEGs. **(A–C)** ssGSEA analysis of the three hub genes (BPGM, DHFR, and SLC25A13) (*p*.adjust <0.05). **(D)** Heatmap of correlations between the three biomarkers (*p < 0.05, **p < 0.01, and ***p < 0.001).

In an attempt to decipher the underlying biological mechanisms of BPGM, DHFR, and SLC25A13 in the development of endometriosis, the three aforementioned biomarkers were analyzed in single-gene GSEA. According to the GSEA assay, it was found that BPGM was actively involved in some biological processes, and the top five most significantly enriched results are shown in Figure 8A. They were “PROTEIN_EXPORT,” “PROTEASOME,” “NUCLEOTIDE_EXCISION_REPAIR,” “MISMATCH_REPAIR,” and “RNA_DEGRADATION.” For both DHFR and SLC25A13, the top five features they were enriched for were completely consistent, namely, MISMATCH_REPAIR, DNA_REPLICATION, PROTEIN_EXPORT, NUCLEOTIDE_EXCISION_REPAIR, and PROPANOATE_METABOLISM (Figures 8B, C). The foregoing findings indicated that these three biomarkers contribute to endometriosis progression through gene expression regulation, protein translocation, and propanoate metabolism. To explore the association of expression patterns between BPGM, DHFR, and SLC25A13, the correlation among them was analyzed. The results of gene correlation analysis demonstrated that there were high associations between BPGM and SLC25A13 (R = 0.77) and DHFR and SLC25A13 (R = 0.75), with a relatively lesser but still positive correlation between BPGM and DHFR (R = 0.53) (Figure 8D). It disclosed the synergistic roles of the three aforementioned core genes in cellular metabolism, proliferation, and disease genesis.

### 3.7 Clinical diagnostic potency of the lactate-related genes model

**FIGURE 9:**
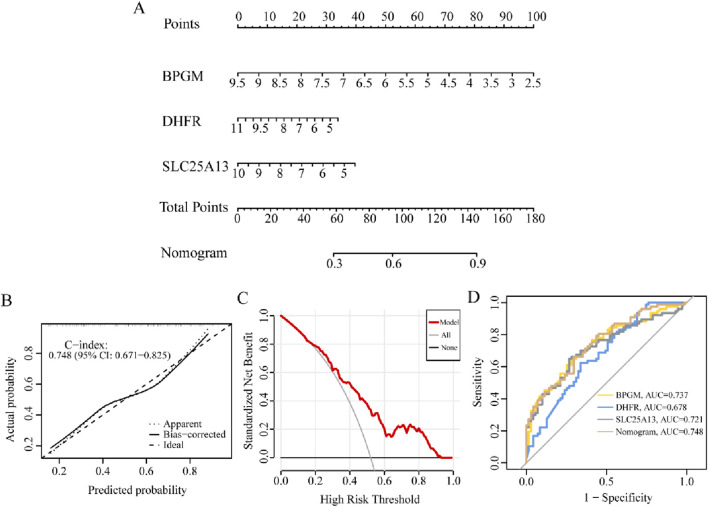
Diagnostic performance analysis for the lactate-related genes model. **(A)** Constructing the nomogram incorporating BPGM, DHFR, and SLC25A13. **(B, C)** Calibration curve and DCA curves for assessing the feasibility of the nomogram model. **(D)** ROC analysis of the nomogram model.

Striving to further research the relationship between the candidate biomarkers and the occurrence risk of endometriosis, the predictive nomogram containing BPGM, DHFR, and SLC25A13 was developed based on the above results, where each gene could be scored individually and summed up to acquire a total predictive score for the possibility of endometriosis (Figure 9A). Then, we have applied the calibration curve and DCA to evaluate the clinical efficacy of the nomogram, and the calibration curve in this study was close to the ideal curve, indicating that the diagnostic potency of the model was favorable, and similarly, the DCA reached the same conclusion (Figures 9B, C). In addition, ROC analysis of three critical genes along with the prediction model in the training set exhibited that the AUC values of BPGM, DHFR, and SLC25A13 were 0.737, 0.678, and 0.721, respectively, whereas the nomogram model achieved the best classification property, with an AUC of 0.748 (Figure 9D). All these results signified that the model possessed superior performance in anticipating endometriosis risk, which can provide a valuable reference for making clinical decisions.

### 3.8 Establishment of lactate-related subtypes and differentiation of the immune microenvironment

**FIGURE 10:**
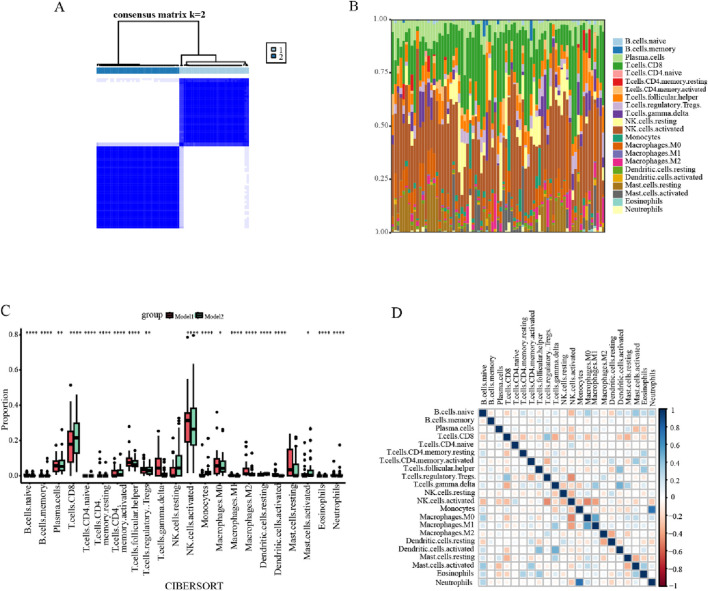
Consensus clustering analysis and immune cell infiltration analysis. **(A)** The clustering heatmap indicated that the clustering results were most stable at K = 2, dividing all endometriosis patients into two subtypes. **(B)** Bar graph representing the infiltration of 22 immune cells in all endometriosis samples. **(C)** Boxplots for the difference in the infiltration abundance of 22 immune cells between models 1 and 2. **(D)** Heatmap displaying the correlations and distributions of 22 immune cells into the two clusters (*p < 0.05, **p < 0.01, ***p < 0.001, and ****p < 0.0001).

To investigate the relationship between the three precious biomarkers and endometriosis subtypes, a consensus clustering analysis was carried out for all endometriosis patients. Based on the consensus matrix and the CDF curves, it was evident that the maximum internal consistency and the minimum external consistency between clusters were attained when k = 2 (Figure 10A; Supplementary Figure S4). Consequently, the total endometriosis samples were categorized into two subtypes, designated as model 1 and model 2. Taking into account that immunological factors play crucial roles in the development of endometriosis, the immune landscape of endometriosis patients was further analyzed. Twenty-two immune cell infiltrations in the two subsets were assessed using the CIBERSORT algorithm. The bar diagram overviewed the distribution of immune cells for each sample, and a subsequent comparative analysis of immune cell distribution between the two clusters revealed significant discrepancies in the proportional representation of various cell types between models 1 and 2 (Figure 10B). In particular, the naive B cells, memory B cells, resting memory CD4^+^ T cells, follicular helper T cells, activated NK cells, M1 macrophages, M2 macrophages, activated dendritic cells, eosinophils, and neutrophils exhibited notably greater ratios in model 1 than in model 2, while in model 2 there were considerably increased percentages of CD8^+^ T cells, naive CD4^+^ T cells, activated memory CD4^+^ T cells, monocytes, and resting dendritic cells (Figure 10C). Afterward, for exploring the cellular interactions, the correlations between the immune cells were investigated, which indicated that monocytes and neutrophils had the strongest positive association, with activated NK cells displaying a prominent negative correlation with both M0 macrophages and regulatory T cells (Figure 10D).

**FIGURE 11:**
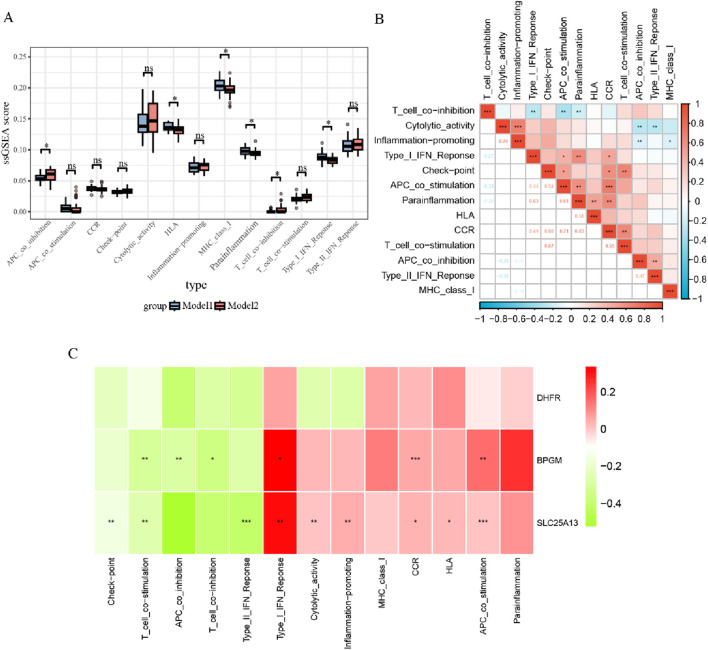
Immune function analysis between two subtypes of endometriosis. **(A)** Boxplots for ssGSEA analysis of immune functions within two subsets. **(B)** Correlation analysis for 13 immune functions in the subgroups. **(C)** Heatmap of correlations between three crucial biomarkers and 13 immune pathways (*p < 0.05, **p < 0.01, and ***p < 0.001).

In addition, the estimate algorithm was applied to evaluate the stromal, immune, and estimation scores for both subtypes, and it was observed that model 2 had higher ratings in all cases, while these three scores were negatively correlated with DHFR and SLC25A13 and positively associated with BPGM (Supplementary Figure S5). To further probe the differences across immune-related function allocation in diverse groupings, the ssGSEA algorithm was performed on model 1 and model 2. The results of immune function analysis revealed that APC co-inhibition, HLA, MHC class I, para-inflammation, T-cell co-inhibition, and type I IFN response were statistically distinct between both cohorts (Figure 11A). Furthermore, the relevance was investigated among various immuno-functional categories. Of these, the most pronounced positive correlation existed between APC co-stimulation and CCR; meanwhile, APC co-stimulation showed the most negative association with T-cell co-inhibition (Figure 11B). Additionally, gene-function correlation analysis identified that type I IFN response, CCR, HLA, and APC co-stimulation were positively correlated with the three core biomarkers (Figure 11C). All of these findings suggested that the three lactate-related genes could contribute to the development of endometriosis through immune mechanisms.

### 3.9 Construction of regulatory networks and prediction for drugs targeting key genes

**FIGURE 12:**
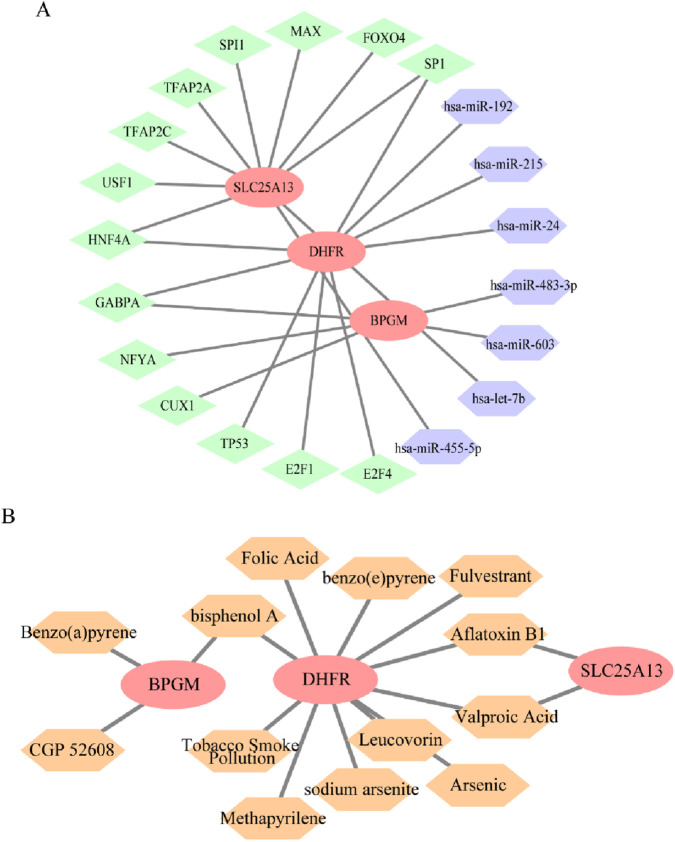
Regulatory network construction and small-molecule drug prediction based on three biomarkers. **(A)** miRNA–mRNA–TF regulatory network of BPGM, DHFR, and SLC25A13, where red represents mRNAs, green represents TFs, and purple denotes miRNA. **(B)** Small-molecule drugs targeting defined genes. Red represents the target genes, and orange represents the drugs.

Transcription factors, miRNAs, and mRNAs interact with each other in sophisticated gene regulatory networks, which affect the biological functions of cells. Consequently, an miRNA–mRNA–TF regulatory network for BPGM, DHFR, and SLC25A13 was constructed to comprehend the mechanism of expression modulation and their functions in disease onset and progression. Notably, SP1 and HNF4A could mediate DHFR and SLC25A13 simultaneously, with GABPA acting on both BPGM and DHFR; in parallel, hsa-miR-192 and hsa-miR-455-5p appeared to influence cell metabolism by regulating DHFR and SLC25A13 (Figure 12A). The linkages between drugs and core genes were further analyzed to recognize potential drug targets for treating endometriosis. Through the CTD database, this study determined three medicines aiming at BPGM, 11 drugs affiliated with DHFR, and two small-molecule agents acting on SLC25A1, which involved multiple biological processes (Figure 12B). One of them, folic acid, a common form of vitamin B, potentially contributes by influencing nutrient metabolism.

## 4 Discussion

Although endometriosis is a common benign inflammatory gynecologic disease, it has biological properties comparable to those of cancer, such as invasiveness and migration, advancement of limited and remote lesions, and resistance to apoptosis (Zhang et al., 2022). Up to this point, no pathogenesis has been proposed that can completely elaborate on the full pathological features of endometriosis, which has compelled several researchers to constantly strive to unearth the underlying mechanisms of endometriosis and achieve early diagnosis and curative treatment by addressing its root cause. Lactate acts as the intermediate of glycolysis, which can participate throughout the entire system in metabolic regulation by functioning as a signaling molecule, and it is an indispensable substance for a variety of physiological cellular functions, exerting pivotal effects in diverse aspects of energy metabolism and signal transduction; meanwhile, in pathological situations, the acid accumulation of lactate in tissue microenvironments represents a characteristic feature of inflammatory diseases and cancers (Certo et al., 2021; Li et al., 2022; Li et al., 2025). Lactylation constitutes an imperative element of lactate function and is involved in tumor proliferation, neural excitability, inflammation, and other biological processes (Li et al., 2022). However, few available research studies have concentrated on endometriosis, the inflammatory disease, and lactate metabolism relevant to it; therefore, we have focused on these two fields, construing the link between them and striving to investigate the pathological features and biological behaviors of endometriosis from the viewpoint of lactate metabolism.

To start with, we screened the RNA expression datasets of endometriosis from the GEO public databases that were appropriate for follow-up analyses; then, we strictly filtered those datasets by layers through the contemporary bioinformatics analytical techniques, and, in combination with the machine learning approaches, we ultimately developed a diagnostic model constructed by BPGM, DHFR, and SLC25A13. BPGM is originally recognized as being uniquely expressed in erythrocytes, where it facilitates oxygen delivery to tissues via lowering the affinity of oxygen in hemoglobin in response to the action of 2,3-bisphosphoglycerate (Oslund et al., 2017; Kulow et al., 2025). With advancing investigations, it has been discovered that one of the detected biological functions of BPGM is its ability to govern the intermediate levels of glycolysis, which in turn regulates the biosynthetic flux of serine (Oslund et al., 2017). Furthermore, BPGM plays a pivotal role in maintaining glucose metabolism in distant kidney units, whose deficiency is associated with metabolic disequilibrium, enhanced oxidative stress, inflammation, and, eventually, renal impairment (Kulow et al., 2025). Hence, we deduced that differentially expressed BPGM in endometriosis promoted metabolic reprogramming by influencing glycolysis levels in ectopic lesions, thus enabling their survival in new environments and subsequent proliferation to develop ectopic foci. DHFR has emerged as a critical enzyme in the metabolism of folate, where inhibition induces deficiencies in active folate, which in turn disrupts nucleotide biosynthesis and subsequently contributes to cell death, thereby constituting an effective target for disease treatment (Sehrawat et al., 2024; Wang et al., 2024). Although there are currently available technological methods that enable DHFR inhibitors in the treatment of diseases, including rheumatoid arthritis, tuberculosis, malaria, toxoplasmosis, and tumors, neither the effect nor the function of DHFR in endometriosis has yet been demonstrated in the literature (Aftab et al., 2024). Serving as the largest solute transporter family in humans, the SLC25 mitochondrial carrier family bridges cytoplasmic and mitochondrial metabolic pathways to underpin cellular and mitochondrial growing and sustaining activities (Cimadamore-Werthein et al., 2024). The protein encoded by SLC25A13, a member of this family, is a calcium-binding aspartate–glutamate carrier protein, alternatively known as citrin, and the mutation of the SLC25A13 gene is responsible for the deficit of citrin, triggering the development of autosomal recessive hereditary metabolic liver disease, which manifests predominantly as intrahepatic cholestasis and assorted metabolic derangements, and in the most serious cases, it progresses to hepatic failure (Inui et al., 2024; Kido et al., 2024). It is expected that our study has first revealed the connection between endometriosis and the above three genes and comprehensively unveiled the novel biological functions and pathological significance of BPGM, DHFR, and SLC25A13 from the viewpoint of lactate metabolism.

In addition to the excellent performance of these three core genes in terms of biological functions, they also demonstrate outstanding values in the diagnosis of endometriosis, and all of them have high AUC levels; simultaneously, the model constructed on their basis represents the highest AUC value of 0.748, which indicates that the signature manifests an incomparable role in the effectiveness of the endometriosis diagnosis. This model, as a means of early diagnosis in the clinical management of endometriosis patients, can assist in the timely recognition of this disease when the typical lesions of endometriosis have not been developed and the corresponding concomitant clinical manifestations have not reached seriousness, and it can offer therapies in the initial stage of illness, which will minimize the alteration of pelvic anatomical structures caused by endometriosis, attenuate the destructive effects of lesions on the ovaries, decrease the incidence of endometriosis-associated infertility, and contribute to the protection of the fertility in the individuals with endometriosis.

Following this, we performed consensus clustering analysis to probe the roles and values of this model in the context of the immunity of endometriosis and split this training dataset into two clusters. Through the CIBERSORT algorithm, we observed that each sample of the two subtypes is well-characterized by a wide range of immune cell types, encompassing most of them in all samples, while the immune cells are scattered among each individual in the different subgroups, which reflects that the immune cells in endometriosis are both generalized and heterogeneous. In addition, this observation is also reinforced by the research of Jae-Wook Jeong, who identified that the characteristic of endometriosis is the dysfunction of the immune system (Rahman et al., 2025). Furthermore, regarding the comparison of the proportion of immune cells, we have pointed out that both subgroups display obvious distributional differences in 19 out of 22 immune cells, which implies that we can formulate personalized endometriosis treatment programs tailored to specific immune cell distinctions based on the model, and this will improve the efficacy of treatment and mitigate the side effects of the conventional medicines on the normal tissues. In the boxplot of immune cell distribution, we found that the NK cells account for a higher percentage in endometriosis, and on comparing two groups of model 1 and model 2, we noticed that the proportion of activated NK cells is greater in model 1 than in model 2, and the ratio of resting NK cells is smaller in model 1 than in model 2. With the role of immunosurveillance and the ability of exerting cytotoxicity of NK cells in endometriosis, the NK cells can assist in the clearance of ectopic endometrial lesions, and we can recognize and promote the activated state transformation of NK cells of model 2 based on the characteristics of this subtype to augment their immunoclearance effects and achieve the clinical treatment of endometriosis. Moreover, activated NK cells and Treg cells presented a strong negative correlation in the immune correlation heatmap, which is in accordance with the previous studies on immune characteristics of endometriosis, where Treg cells have immunosuppressive properties and, together with the abovementioned features of NK cells, cooperate in preventing the adhesion, proliferation, and invasion of ectopic endometriotic lesions (Ohkura and Sakaguchi, 2020).

Aside from examining the discrepancies between the two subgroups regarding immune cells, we have also analyzed the differences in immune function between models 1 and 2. Whether it is model 1 or 2, we can observe in the heatmap of the functional distribution that MHC-class 1 is widely present in endometriosis, and it is known to be associated with the immune escape of the disease, which further confirms that endometriosis is an immunological disease, and this result also supports the accuracy of our analytical strategy (Brea et al., 2016; Blanco et al., 2025). Scores on the evaluation of immune function in terms of APC co-inhibition and T-cell co-inhibition were found to be higher in model 2 than in model 1. CTLA4, as a molecule featured in the function of T-cell co-inhibition, is inherently inhibitory in character and is a key immune regulatory member belonging to the family of type I membrane receptors, with evidence supporting the involvement of CTLA4-based autoimmunity in maintaining chronic inflammation in the peritoneal environment of endometriosis and preclinical samples of evidence indicating anti-CTLA4 antibodies as a prospective novel therapeutic target for endometriosis (Liu et al., 2020; Mikuš et al., 2022). PD-L1, which is a commonly involved factor in the highly salient functional modules of the immune function scoring in model 2, is elevated in endometriosis, and this upregulation suppresses the cytotoxic activity of T and B lymphocytes, thereby reducing their effectiveness in eliminating ectopic endometrial tissues, leading to their immune evasion and bolstering the survival and proliferation of the ectopic lesions (Sobstyl et al., 2024; Nero et al., 2022). These observations propose that protocols based on this lactate-associated gene model subtyping targeting specific molecules of model 2 could be explored as new alternatives for the management of endometriosis.

At the end of this research, we explored the upstream regulatory network of the lactate-related gene model in which transcription factors and miRNAs, as common modulators of gene expression, are involved in the construction of the above network. Specifically, SP1 was identified as a transcription factor that can govern DHFR and SLC25A13 in this network, and previous studies have confirmed that SP1 plays a central role in adjusting the expression level of DHFR, which mainly functions by influencing the cell-cycle progression (Slansky and Farnham, 1996; Blume et al., 1991). After reviewing substantial publications, we were not aware of established roles of SP1 in SLC25A13, and this study offered a compelling new direction for investigating the differential expression of SLC25A13. In terms of subsequent clinical applications, we tried to identify small-molecule drugs targeting this model; valproic acid, as a branched-chained fatty acid, is widely used for the treatment of epilepsy, and at the same time, it also exists as an adjuvant reagent in breast cancer therapy (Davis et al., 1994; French and Faught, 2009; Heers et al., 2018). Our study may have identified an emerging benefit of valproic acid, and in the future, we expect to uncover and validate the value of valproic acid for endometriosis therapy from the aspects of mechanism experiments, molecular assays, and clinical trials.

Regardless of the fundamental contribution of our lactate-related gene signatures in predicting early diagnosis and guiding the treatment of endometriosis, there are some limitations to this research. First, although both the training and validation groups were available in this study, the analysis was performed based on the sample information in the database, and it has not been carried out in large-scale, multicenter, prospective cohort studies, nor has it been subjected to more detailed stratified analysis incorporating clinical phenotypic characteristics. Furthermore, we will proceed to explore this model with *in vitro* and *in vivo* experiments and molecular mechanism studies if necessary to find the molecular mechanisms of lactate metabolism in endometriosis and generate supporting data for the development of targeted molecular therapies. Finally, despite the fact that our study constructed the regulatory network of transcription factors and targeting drugs within the upstream of the model, the working potency of the networks and the specific sites and the safety of pharmacological action still require further exploration.

## 5 Conclusion

In general, this study has illustrated the relationship between lactate metabolism and endometriosis, in which we constructed a lactate-associated gene model comprising three genes: BPGM, DHFR and SLC25A13, which serve invaluable functions in the diagnosis of endometriosis and enable early recognition of the disease. Simultaneously, we also carried out the analysis of the immunological aspects of endometriosis on the basis of this model in an attempt to detect potential therapeutic approaches for targeting the development of endometriosis from the perspective of immunotherapy. Beyond that, we have established the crucial gene regulatory network to characterize the interactions of networks in a multidimensional and comprehensive manner to filter the innovative medical agents for the prevention and treatment of endometriosis from the viewpoint of small-molecule compounds. In conclusion, our research aimed to find the lactate metabolism-related pathogenesis of endometriosis, construct an early diagnostic model, and implement accurate targeted prevention and clinical management for patients with endometriosis.

## Data Availability

The datasets presented in this study can be found in online repositories. The names of the repository/repositories and accession number(s) can be found in the article/Supplementary Material.
