# Supplementary material for: Identification and validation of lactate-related gene signatures in endometriosis for clinical evaluation and immune characterization by WGCNA and machine learning
Source: Front Cell Dev Biol. 2025 Oct 7;13:1672521. doi: 10.3389/fcell.2025.1672521 (PMC12537695; doi:10.3389/fcell.2025.1672521)
Supplement: Supplementary file 1 [file Presentation1.pdf]

1 Supplementary material

Supplement Table S1 The primer sequences of 3 LR-DEGs and Actin

Gene name		Sequence of primer (5'- 3')
BPGM	Forward	AACTCAACAGCGAAGGAATGG
	Reverse	AATGGACCGATTAAGGACAGATG
DHFR	Forward	TTCCAGAGAATGACCACAACCT
	Reverse	GCCACCAACTATCCAGACCAT
SLC25A13	Forward	ACTGCGTGAAGAAGGACCAA
	Reverse	GGTAGATACTGATGGCTTGAAGAG
Actin	Forward	AGCGAGCATCCCCCAAAGTT
	Reverse	GGGCACGAAGGCTCATCATT

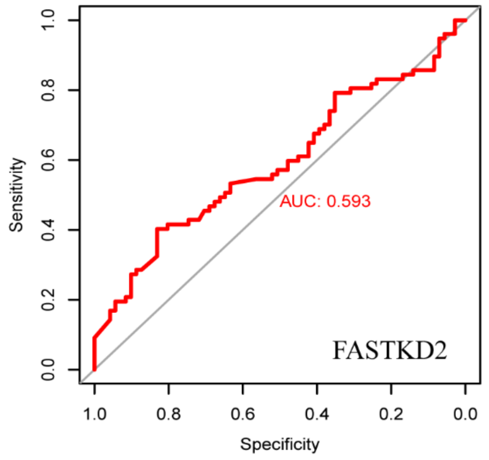

Figure S1 The ROC analysis for FASTKD2 in the training dataset.

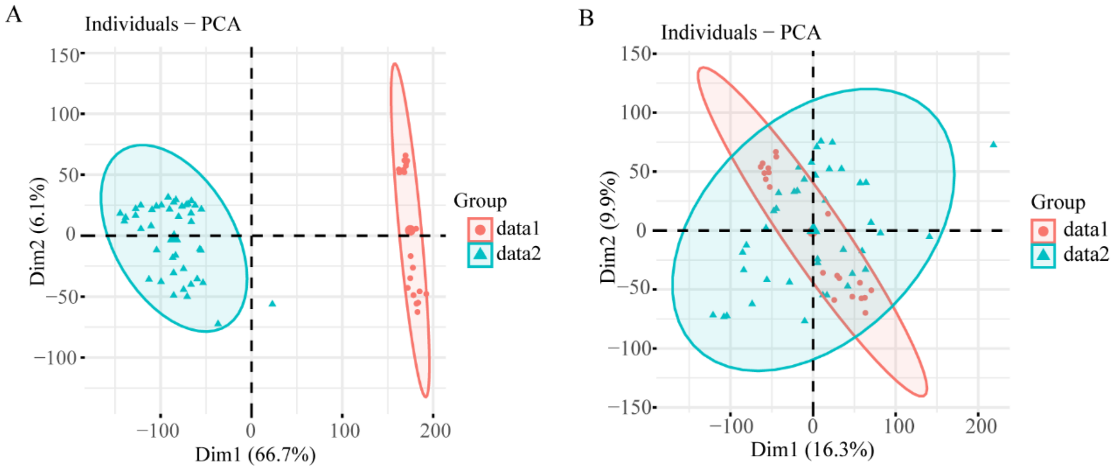

Figure S2 Combining the GSE7305 and GSE7307 datasets. A, PCA of samples from the two databases before data merging. **B,** PCA plots for the both datasets after eliminating the batch effect.

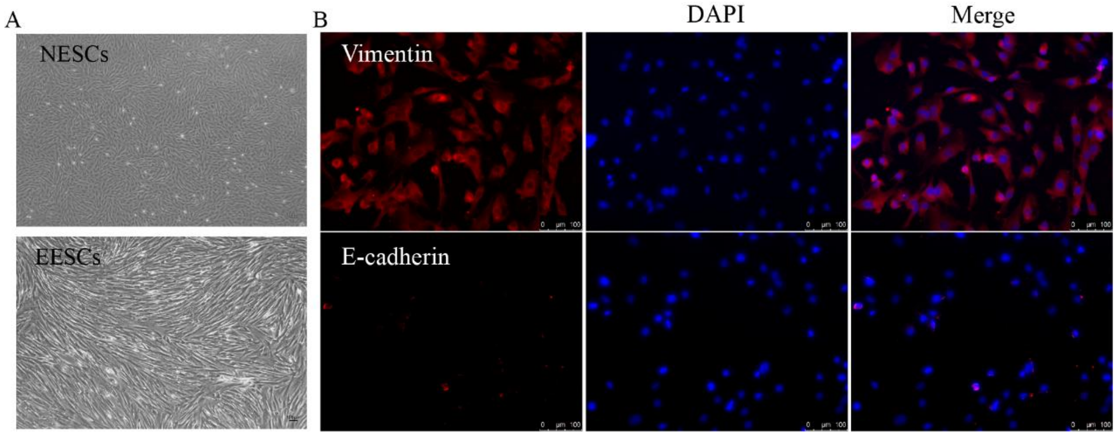

Figure S3 The characterization of endometrial stromal cells. A, The morphology of primary endometrial stromal cells. **B,** Identification of endometrial stromal cells by immunofluorescence.

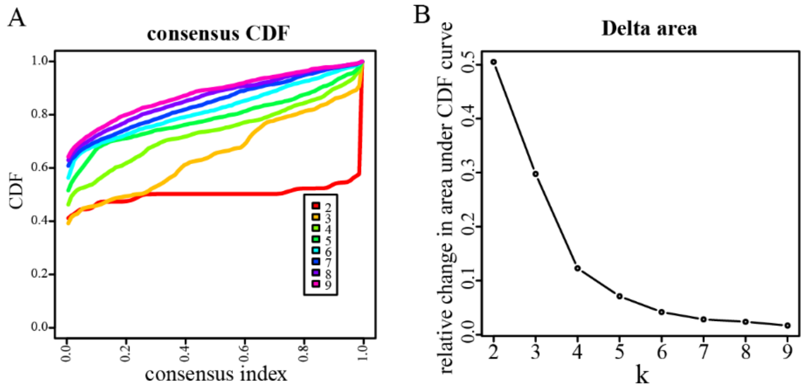

Figure S4 Results of consensus clustering analysis. A-B, CDF curves and Delta area curves indicating that the clustering results were most stable at K=2.

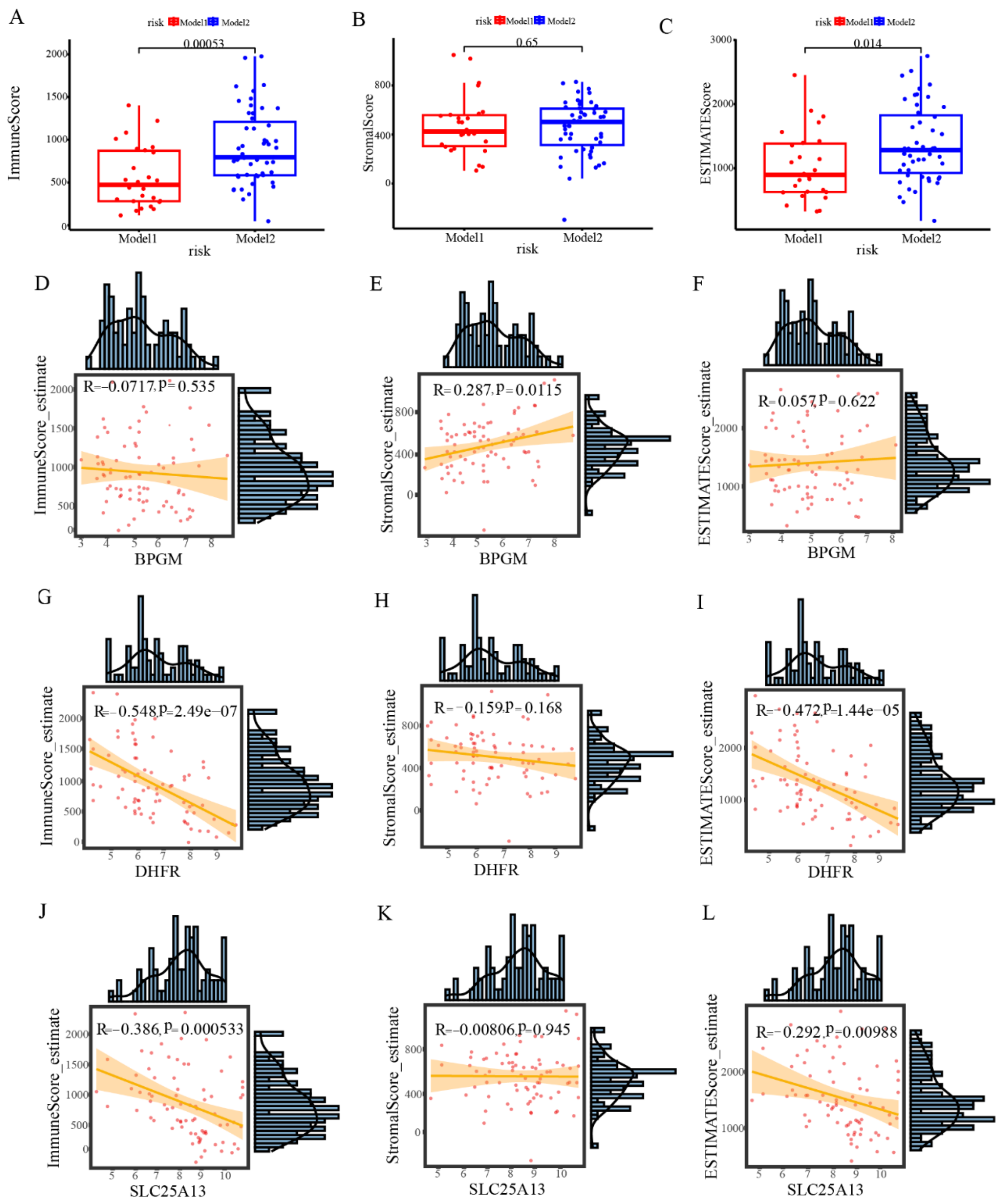

Figure S5 ESTIMATE algorithm evaluating the effect of DHFR, BPGM and SLC25A13 on the immune microenvironment. A-C, Box plots indicating the Immune, Stromal and Estimate scores between the two clusters. **D-F,** Scatterplot showing correlation analysis between BPGM and Immune, Stromal, and ESTIMATE scores. **G-I,** The correlation analysis between DHFR and Immune, Stromal, and ESTIMATE scores. **J-M,** The scatter plots displaying the relevance of the SLC25A13 to the Immune, Stromal, and ESTIMATE scores.
